# Impact of Processing Conditions on Inter-tablet Coating Thickness Variations Measured by Terahertz In-Line Sensing

**DOI:** 10.1002/jps.24503

**Published:** 2015-06-02

**Authors:** Hungyen Lin, Robert K May, Michael J Evans, Shuncong Zhong, Lynn F Gladden, Yaochun Shen, J Axel Zeitler

**Affiliations:** 1 Department of Chemical Engineering and Biotechnology, University of CambridgeCambridge, CB2 3RA, UK; 2 TeraView Ltd, St John's Innovation ParkCambridge, CB4 0DS, UK; 3 Department of Electrical Engineering and Electronics, University of LiverpoolLiverpool, L69 3GJ, UK

**Keywords:** coating, imaging methods, physical characterization, process analytical technology (PAT), quality by design (QBD), tablet, Processing, Unit operations

## Abstract

A novel in-line technique utilising pulsed terahertz radiation for direct measurement of the film coating thickness of individual tablets during the coating process was previously developed and demonstrated on a production-scale coater. Here, we use this technique to monitor the evolution of tablet film coating thickness and its inter-tablet variability during the coating process under a number of different process conditions that have been purposefully induced in the production-scale coating process. The changes that were introduced to the coating process include removing the baffles from the coater, adding uncoated tablets to the running process, halting the drum, blockage of spray guns and changes to the spray rate. The terahertz sensor was able to pick up the resulting changes in average coating thickness in the coating drum and we report the impact of these process changes on the resulting coating quality. © 2015 The Authors. *Journal of Pharmaceutical Sciences* published by Wiley Periodicals, Inc. and the American Pharmacists Association J Pharm Sci 104:2513–2522, 2015

## INTRODUCTION

The process of applying one or more layers of polymer coating onto tablets is almost ubiquitous in pharmaceutical manufacturing in order to simultaneously achieve uniformity of colour, light protection, taste masking and, more recently, to control drug release kinetics and thereby increase the therapeutic efficacy of tablets.^1^ Tablet coating is typically performed in large batches, and the quality of the resulting product is reflected in the intricacies of the tablet mixing dynamics which in turn is dependent on tablet properties (e.g. size and shape), process parameters (e.g. coating pan speed and loading) as well as device specific parameters (e.g. pan diameter, geometry, baffle configuration etc.). In addition to average coating thickness, other metrics that govern the quality of the finished product include intra- and inter-tablet coating uniformity, surface roughness and structural integrity of both the coating and the tablet core.

Various approaches have been employed to gain a better understanding of the complex relationships that exist between the many factors that ultimately determine the coating quality. Recently, there has been a significant drive towards systematic process understanding by means of statistical design of experiments as part of the quality-by-design framework that takes account of the manufacturing process from development through to scale-up.^2^ Although feasible at the development stage, experimental studies made at the production scale are costly and wasteful. An alternative is to undertake rigorous numerical simulation, which has demonstrated tremendous potential for improving the understanding of film coating processes.^3^–^8^ Such models can predict both inter- and intra-tablet coating variations; however, the accuracy of predictions are subject to the accuracy of the input data, which are often estimates based on assumptions as opposed to the results of measurements made under actual process conditions.^9^ In an effort to provide experimental feedback on the coating process, and for possible subsequent process control, different sensor technologies have been introduced to non-destructively measure the tablet coating thickness either in-line or on-line. Examples of such techniques include optical sensing at near-infrared frequencies^10^ and Raman spectroscopy.^11^–^13^ Comprehensive reviews on the topic have been previously published.^14^,^15^ Typically, these rapid sensing techniques monitor the spectral attenuation of chemical constituents within the dosage forms directly, from which the average thickness of sampled dosage forms can be inferred using previously created multivariate calibration models. Although these techniques can determine process end-points as well as moisture content that would be important for process control, the calibration models are time-consuming to construct, require ongoing maintenance support and provide prediction performances that are specific to the instrument. Even in cases of the same vendor and model, the transferability of the models is not always seamless and often the models must be reconstructed. Furthermore, the measurements acquired are a time-averaged result over the numerous sampled dosage forms and therefore information pertaining to the individual dosage form, such as inter-tablet coating uniformity, is simply unavailable. Other techniques such as optical imaging^16^ which, as well as providing tremendous throughput with the use of modern visual imaging systems that reduces the equipment cost barrier, can sample individual tablets and therefore provide information on inter-tablet coating uniformity. However, based on the authors' knowledge, optical imaging techniques are currently limited in application to monitoring of spherical dosage forms, largely because of the simplicity involved in determining the coating thickness. Tablets with complex shapes may therefore pose a challenge to thickness calculation. Spectral-domain optical coherence tomography is a recently introduced modality for direct coating thickness measurement of individual dosage forms that boasts high spatial resolution, both laterally and axially^17^,^18^; therefore, making it ideal for the measurement of thin coatings. Although the technique is promising, especially given its high data acquisition rate, thus yielding inter- and intra-tablet coating uniformity information, the technique is still very much in its infancy, thus requiring further research, in particular to assess the maximum thickness coating that can be measured because of the stronger scattering encountered with this technique.

Terahertz pulsed imaging (TPI) was previously demonstrated as a suitable modality to measure the coating thickness of individual pharmaceutical tablets off-line and at-line.^19^ This measurement technique exploits the fact that the common pharmaceutical excipients, being primarily polymer based, are amorphous and (semi-)transparent to radiation at terahertz frequencies. Moreover, the coherent and broadband nature of terahertz radiation makes it possible to readily determine the depth at which sub-surface material interfaces occur. By mounting a terahertz sensor externally onto a perforated coating pan such that the tablets inside the rotating coating pan are kept in focus of a continuous train of terahertz pulses, the time lapse between successive reflections from coating interfaces can be measured directly to determine the coating thickness of individual tablets. In order to determine the absolute coating thickness, however, knowledge of the refractive index of the coating material is required, and such information is readily attainable using terahertz time-domain spectroscopy as outlined previously.^20^ Compared with the aforementioned measurement techniques, terahertz pulsed technology is quite unique in that it can measure the tablet coating thickness directly and can resolve the thickness of a large number of individual tablets inside the production-scale coating pan at any given point in the coating process. Having introduced the in-line terahertz pulsed technique previously,^20^ the objective of this study is to further investigate the ability of the technique to monitor changes in the tablet coating thickness distribution inside a production-scale film coating unit, in the presence of artificially induced variations in the coating process. Although previous studies have investigated changes in inter-tablet coating uniformity as a result of varying process conditions, those studies were conducted at-line or off-line.^21^ In contrast, the present work reports the findings from an in-line investigation conducted in a production-scale setting.

## MATERIALS AND METHODS

An in-line terahertz sensor system (TeraView Ltd., Cambridge, UK) was developed and installed on the side of a production-scale, side-vented perforated pan tablet coater (Premier 200; Oystar Manesty, Merseyside, UK). To ensure that the generated terahertz pulses were focused onto the surface of tablets inside the coating pan, the sensor was kept at a fixed distance (corresponding to the 7 mm focal length of the sensor optics) from the inner wall of the coating pan. The perforated pan had an overall diameter of 1.3 m, whereas each circular perforation had a diameter of 3 mm. The patterning of the perforations resulted in a 51% opening on the external surface of the pan. The coating pan was fitted with tubular baffles to facilitate the mixing of the tablet bed. During the coating trial, a polymer film (Acryl-EZE R, Aqueous AcrylicEntericSystem yellow and/or pink; Colorcon Ltd., Dartford, UK) was applied to each batch of tablets. The batch size of uncoated tablet cores was 175 kg. The tablet geometry was bi-convex (10 mm diameter, 370 mg) and consisted of direct compressed lactose monohydrate (Meggle, Wasserburg, Germany). Coating was performed using three spray guns at a spray rate of 300 mL/min operating at an atomising air pressure of 1.5 bar. The coating pan had a rotational speed of 6 rpm. The inlet air flow was set to 2200 m^3^/h at a temperature of 52°C and an absolute water content of 7.6 g/kg. The exhaust temperature was maintained at 38°C–40°C.

**Figure 1:**
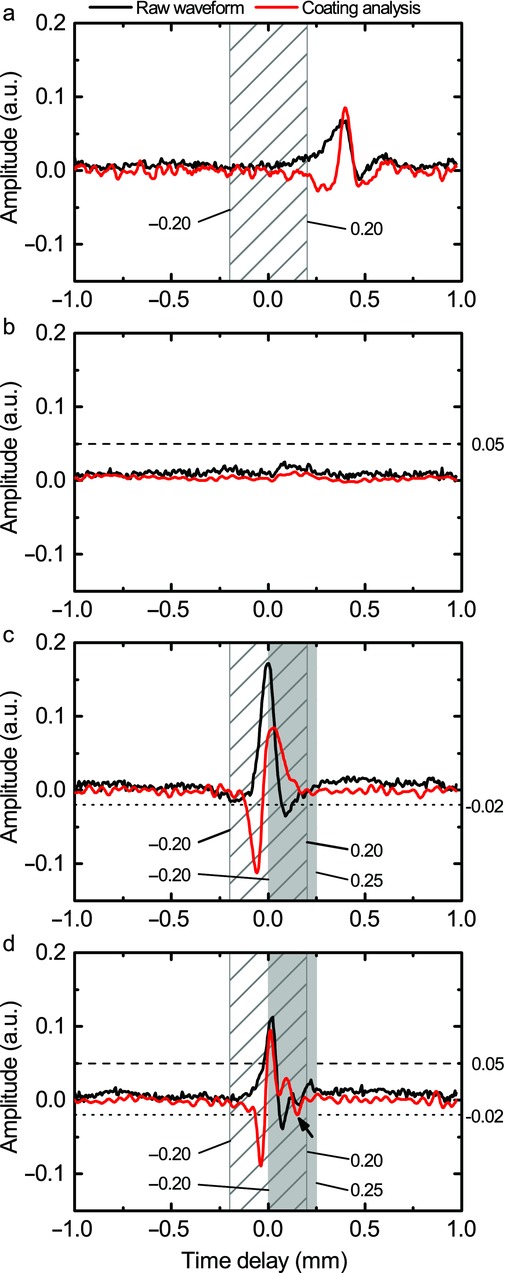
Examples of three rejected waveforms and one accepted waveform. An example of the waveform that failed due to its reflection occurring outside the primary pulse position range (a), waveform not matching the primary pulse amplitude range (b), the secondary pulse amplitude range (c), and an accepted waveform that satisfies all these criteria (d) with an arrow pointing at the secondary peak from the coating-to-core interface.

**Figure 2:**
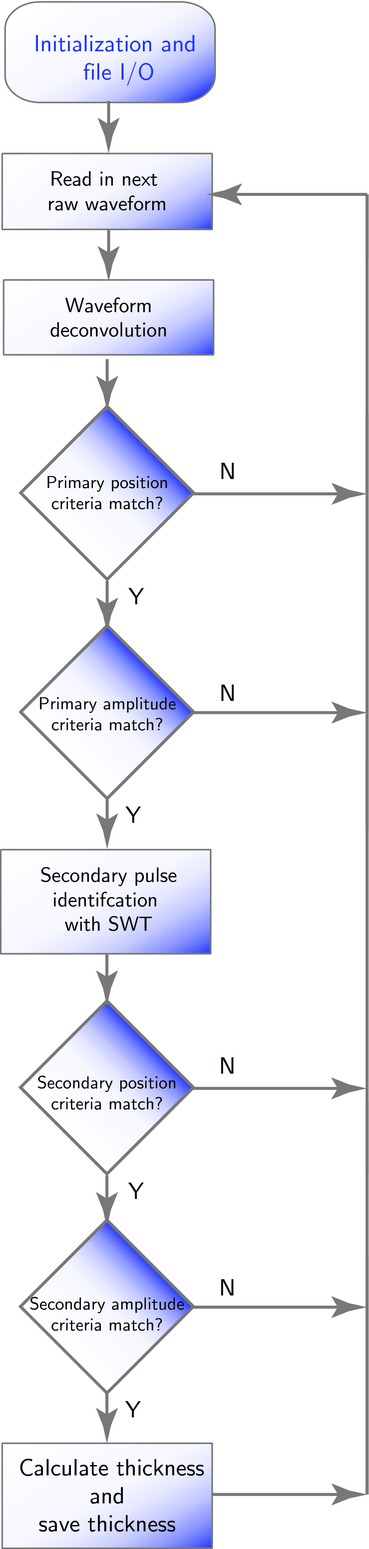
Activity diagram of the data processing algorithm to systematically identify high-quality waveforms for use in coating thickness determination from in-line data acquired and processed in real time.

The installed terahertz in-line sensor continuously acquired individual terahertz waveforms at a rate of 120 Hz, however not every waveform contains reflections from a tablet surface. Examples of the acquired waveforms are shown in Figure 1. Because the circular openings in the perforated coating pan account for about half of the external surface of the coating pan wall that is presented to the sensor head in each rotation of the coating pan, about half of the measured waveforms can contain a reflection that originates from a tablet inside the pan. This number is further reduced because not every aperture will have a tablet directly behind it, nor will all tablets behind an aperture be suitably aligned at normal incidence to the terahertz sensor, the optimum orientation needed to obtain a high-quality measurement. In order to correctly identify those reflected waveforms that are suitable candidates for the subsequent coating thickness calculation, all measured waveforms are automatically processed in real time during data acquisition using the waveform selection algorithm described by the flowchart shown in Figure 2.

Prior to data analysis, signal processing is performed on the raw pulse waveform^19^ in order to isolate the sample response from the system response. Generally, the raw pulse waveform shows reflections that arise from each interface or abrupt change in refractive index encountered by the incident terahertz pulse as it propagates into the sample. The relative strength of the reflections indicates the change in physical or chemical composition at the interface. Scattering losses, because of refractive index changes at grain boundaries, are typically not significant in pharmaceutical dosage forms because of the absence of structure on length scale of hundreds of micrometres (the range of the wavelength at terahertz frequencies) in the coating layers. The first step in signal processing involves performing a waveform deconvolution with a reference waveform obtained from the ideal reflecting surface of the outer metallic mesh wall of the coating pan,^20^ so as to yield time-domain waveforms of a high signal-to-noise ratio that clearly reveal individual reflections from interfaces across which changes in refractive index occur. By applying a set of pre-determined selection criteria to these processed waveforms, only the waveforms that originate from the surface of a coated tablet that is within a range of normal orientation to the terahertz sensor are selected for the subsequent coating thickness calculation. Specifically, the position and amplitude of those reflection peaks of interest contained in a given sample waveform must fall within pre-defined limits, as is illustrated in Figure 1. A tablet with a single coating contains two reflection peaks of interest: the first peak corresponds to a reflection from the air-to-coating interface, and the second peak to a reflection from the coating-to-core interface. The thresholds values are determined from off-line measurements in which reflected waveforms from individual tablets are measured at a series of distances from and angles to the sensor focusing lens, so as to identify a suitable peak position and amplitude ranges within which reliable coating thickness can be determined.

The selection criteria are applied to all the processed sample waveform following the flowchart shown in Figure 2. Each must contain a primary reflection peak and the position and amplitude of which lies within the corresponding limits. Examples of waveforms that are rejected because the primary reflection peak fails to meet these criteria are shown in Figure 1, where the dashed lines and shaded areas depict the position and amplitude thresholds. If the primary reflection peak satisfies the position and amplitude criteria, analysis is performed with stationary wavelet transform to identify the presence of a secondary reflection peak within a realistic pulse delay range (30–200 μm). In particular, Haar wavelets are used with four levels of decomposition as it has proven to be a more robust peak finding method in the time domain.^22^ The amplitude of the secondary pulse within the range must then exceed a certain threshold value for coating thickness to be reliably calculated. An example of a waveform that has been rejected from further analysis because of the second reflection peak having an amplitude below the pre-defined amplitude range is also shown. In contrast, the bottom of Figure 2 shows a suitable waveform that has passed all the selection criteria and thus can be used for coating thickness calculation. As coating thickness is directly proportional to the time lapse between consecutive reflection peaks in the time domain and inversely proportional to the refractive index, the coating thickness *d* is determined as *2d* = Δ*tc*/*n*, where Δ*t* is the time lapse, *n* is the coating refractive and *c* is the speed of light in vacuum. In this particular example, the measurement resulted with a coating thickness of 87.6 μm for a coating refractive index of 1.55.

The particular values assigned to the reflection peak position and amplitude limits must be carefully chosen so as maximise the number of measured tablets while ensuring that only high-quality waveforms are accepted so as to omit low-confidence coating thickness readings. Using the most stringent values for the selection criteria to ensure acceptance of high-quality waveforms only, a tablet hit rate of 8200 (∼0.3% of all measured waveforms) over 6 h of tablet coating was achieved, which corresponds to a ‘hit rate’ of over 20 individual tablets per minute. For a single-coating run with steadily increasing coating thickness, a value of *R*^2^ = 0.91 and a root mean squared error (RMSE) = 5.8 μm were determined for this set of processing parameters when correlated with off-line terahertz thickness measurements made on coated tablets removed at regular intervals during the coating process. In order to optimise the threshold values used by the selection criteria, while taking account of possible experimental uncertainties and the non-concentric nature of the coating pan, a systematic study was conducted to optimise the hit rate. Specifically, we generated a set of possible values for the selection parameters and then tested them when analysing the data from a single-tablet coating run. The optimal values for the various selection thresholds were determined by using numerical optimisation to maximise the number of measured tablets whilst simultaneously maximising the agreement between on-line and off-line thickness measurements (in terms of *R*^2^ and RMSE values). To speed up computation, different selection criteria were applied to the acquired waveforms in parallel on a cluster of four workstations under the Matlab Parallel Computing Toolbox environment (Matlab R2012; The MathWorks Inc., Natick, Massachusetts).

The optimal selection criteria that were identified from this run were subsequently applied to the experimental measurements acquired from a number of coating runs where process variations were artificially induced into the coating process. These variations include the removal and insertion of mixing baffles, the addition of uncoated tablet cores into the pan at a later process time point and altering the coating spray rates during the coating process.

## RESULTS AND DISCUSSIONS

By using the optimal selection criteria, the number of total tablet hits can be increased from 8200 to 16,660, resulting in a value of *R*^2^ = 0.8 and RMSE = 10 μm, a notable decrease in *R*^2^ value that produces significant improvement in the hit rate. Despite the reduction in *R*^2^ value, data quality is generally not compromised; however, we note the introduction of artefacts (thickness ∼150 μm) present in the coating thickness distributions. The strongest influence on the hit rate was found to be the selection criterion for the primary reflection (air-to-coat interface). This can be explained by the fact that the coating pan is not perfectly concentric, thus causing subtle and systematic changes to the position of the measured terahertz waveform in every rotation of the pan. As a result of relaxing the selection criteria for the location of the first reflection peak to account for the concentricity imperfections of the coating pan, additional reflections are considered for thickness calculation. The study to determine the optimal selection criteria took approximately 1 week with two workers on the cluster. This time could be significantly reduced by using a factorial design with a reduced number of combinations and by performing the parallelisation of the code that can be executed on the graphics processing unit (GPU) rather than the central processing unit (CPU).

**Figure 3:**
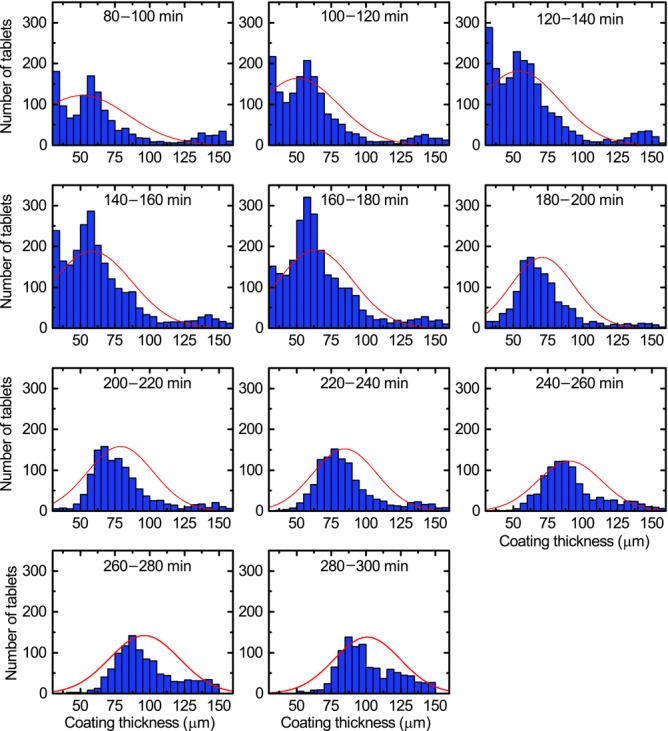
Histogram of tablet coating thicknesses inside the coating pan from 80 to 320 min for the previously published coating run.^20^ The large thickness values (>150 μm) acquired does not represent a reliable measurement and is an artefact because of relaxed acceptance criteria.

**Figure 4:**
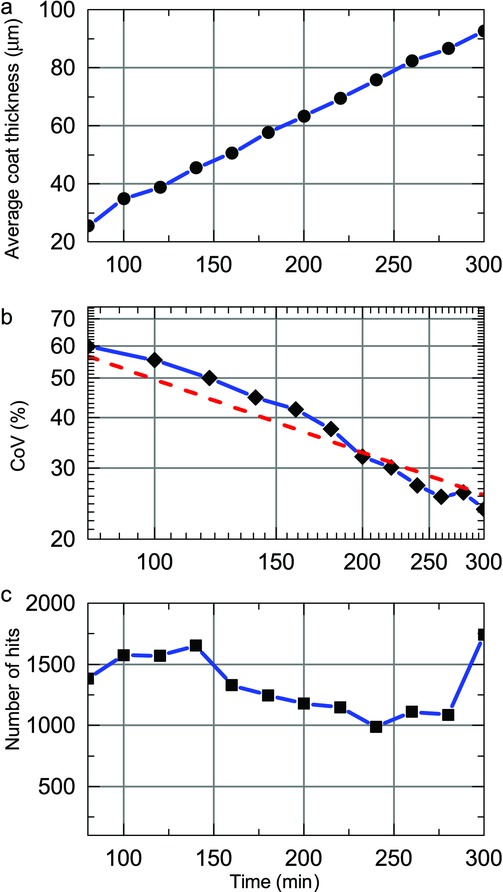
Curve-fitted mean (a) and inter-tablet variability (log–log scale) (b), as well as the number of coated tablet thickness measurements (c) for a previously published coating run.^20^ The linear decrease in the inter-tablet variability is curve fitted with a red dashed line to extract the rate of decrease.

By using the optimal selection criteria on the acquired waveforms it is possible to maximise the amount of tablet coating thickness data that can be extracted from the process in order to analyse the effect of process changes on the inter-tablet coating thickness distribution. Figure 3 shows distributions of tablet coating thickness from data acquired over 20 min process periods (using histogram bin widths of 5 μm) for the previously published coating run.^20^ During the first 60–80 min of the process, the coating thickness is below the minimum resolvable thickness.^20^ Assuming a normal distribution for coating thickness distribution in each time window, the mean and variance of each distribution were estimated by fitting, in a least-squares sense, a Gaussian profile to each histogram. Because all captured reflections may not originate from tablet surface or the centre band, the data may be better described with alternative distributions such as *F*, Chi or Rayleigh distributions. Further work will aim to better discriminate the reflections from the tablet surface and from the centre band. In general, the underlying distribution would be probabilistically dependent on additional parameters not limited to tablet geometry, including the loading level of the coating pan and the rotational speed. Nevertheless, Figure 4 shows the curve-fitted mean and the coefficient of variation (CoV) or the inter-tablet coating thicknesses variability with respect to coating time. Note that the CoV is determined using the relative SD (in %) as opposed to the absolute SD in micrometres. As a number of studies have predicted the change in CoV values during the coating process using the discrete element method (DEM)^3^,^4^,^23^ based on residence time distributions, the CoV of the present experimental study was plotted on a log–log scale for direct comparison with such studies. For our data, the decrease in CoV can be fitted with a straight line with a slope of −0.57, slightly higher in magnitude than the reported value of −0.5, which was found to describe the coating behaviour of tablets on a laboratory-scale coater.^3^ Despite this overall agreement in the relative change of CoV with theoretical modelling the absolute values of the CoV are approximately an order of magnitude greater than those of the CoV measured by off-line imaging.^21^ The high variability observed in our experiments is in agreement with the findings reported in the previously published coating run^20^ and can be explained by the fact that two orders of magnitude more tablets were sampled in the in-line analysis compared with the off-line analysis.

### Effect of Removing Coater Baffles

**Figure 5:**
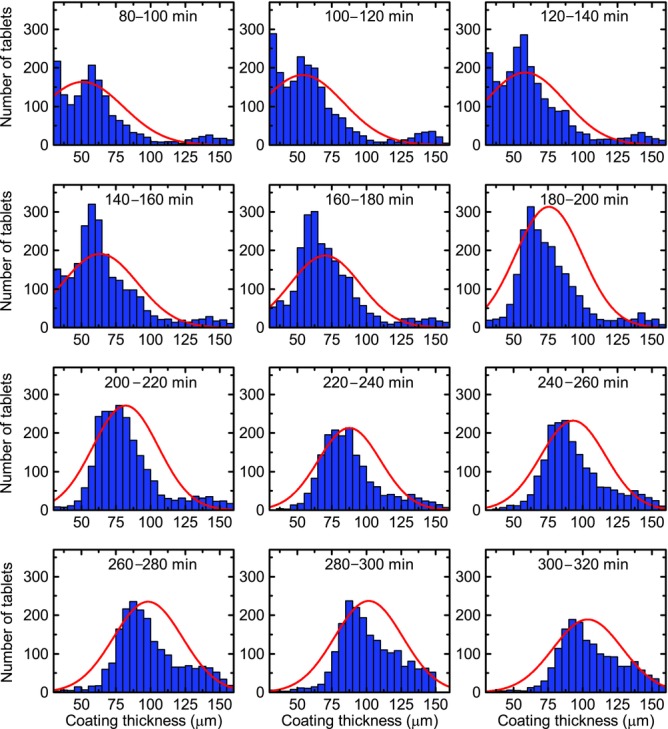
Histogram of tablet coating thicknesses inside the coating pan from 80 to 320 min where the mixing baffles were removed after 200 min. The large thickness values (>150 μm) acquired do not represent reliable measurements and are artefacts because of relaxed acceptance criteria.

**Figure 6:**
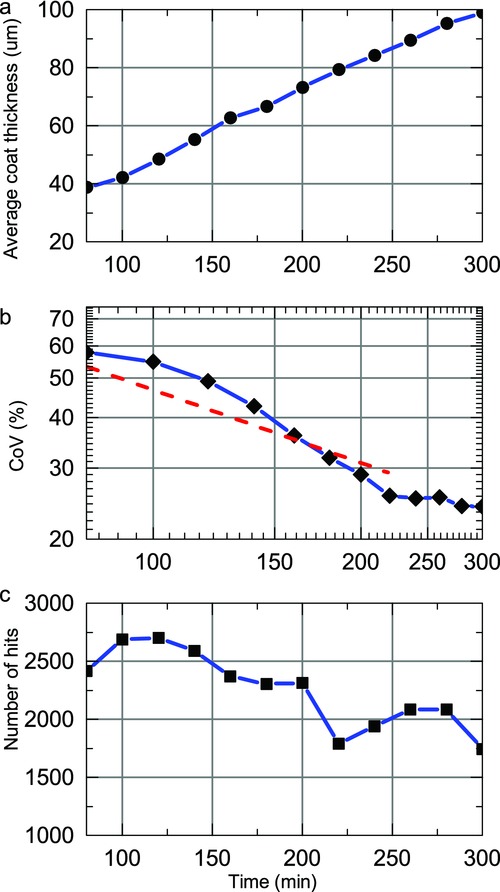
Curve-fitted mean (a), inter-tablet variability (log–log scale) (b) and number of tablet coating thickness (c) during a coating run where the baffles were removed after 200 min process time. Lines are plotted to guide the eye. The red dashed line corresponds to a linear fit describing the decrease in the inter-tablet variability to extract the rate of decrease in CoV.

Figure 5 shows the tablet coating thickness distributions over 20 min periods for a coating run in which the mixing baffles were removed after 200 min of coating. Figure 6 shows the corresponding curve-fitted mean and the CoV as a function of coating time. After 80 min of coating time we observe a monotonic increase in tablet coating thickness. The inter-tablet coating thickness variability also decreases monotonically until just after 200 min of process time (about 220 min). From the time the mixing baffles are removed, the CoV does not decrease further but remains constant at around 25%. It is interesting to note that the mean coating thickness appears to be unaffected by the removal of the baffles. The decrease in CoV up to the point where the mixing baffles have been removed is fitted to a straight line with a slope −0.61, similar to the value of −0.57 obtained for the previous coating run in Figure 4.^20^ Even though the slope is slightly higher in magnitude than the previously reported^3^ value of −0.5, considering the many discrepancies that exist between the parameters used in the DEM simulation and the actual experimental conditions this value is in surprisingly close agreement. The main differences lie in the scale of the present operating conditions compared with those of the simulation: the coater diameter (1.3 m compared with 0.62 m), tablet load (473,000 tablets compared with 22,500) and the number of spray nozzles (three compared with one). All of these factors will affect the tablet residence time within the spray zone. It should also be highlighted that measurement uncertainties were inadvertently introduced by relaxing the selection criteria to overcome the concentricity imperfections of the coating pan. The new selection criteria nonetheless produced a relatively steady hit rate throughout the process, as shown in Figure 6, that was necessary for the process investigation.

### Addition of Uncoated Cores During the Coating Process

**Figure 7:**
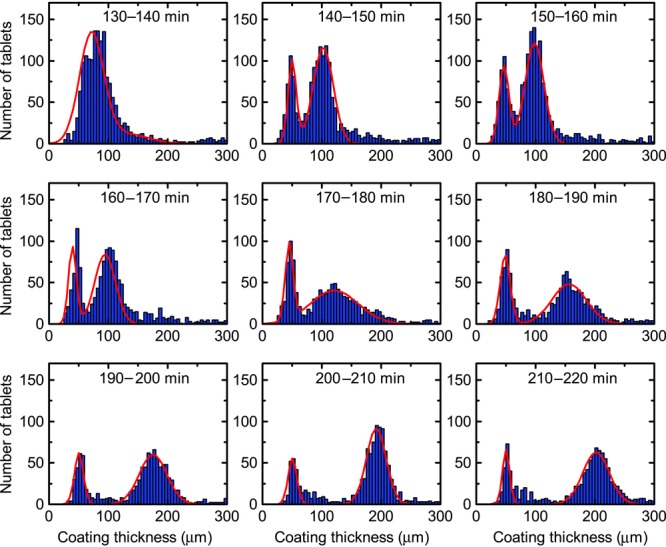
Histogram of tablet coating thicknesses from 130 to 220 min process time with uncoated tablets introduced close to 140 min of the coating process.

**Figure 8:**
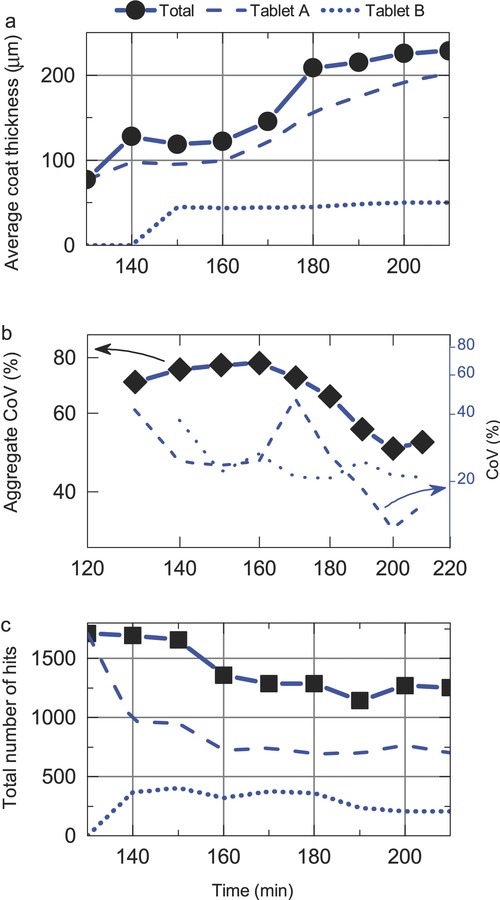
Curve-fitted mean (a), inter-tablet variability based on the width of the Gaussian approximated distribution (log–log scale) (b) and the number of hits (c) of the total population, Tablets A and B (uncoated tablets) were introduced close to 140 min of the coating process.

In another coating run, 87.5 kg of uncoated tablet cores (Tablets B) were added to the coating pan at approximately 140 min into the coating process of an initial batch of the same size (Tablets A). Coating was applied to the combined batches for a further 80 min for a total coating time of 220 min. Figure 7 shows the resulting changes to the coating thickness distributions. The emergence of a second distinct coating thickness distribution representing the additional batch ‘Tablets B’ is clearly visible from 140 min onwards. At the same time, there is a clear shift in the original coating distribution implying continued increasing thickness in the coating of Tablets A. The width and CoV of the two distributions of Tablets A and B (again approximated using a Gaussian function) are plotted in Figure 8. The plot of CoV over the entire coating trial shows that the overall coating thickness variability increases following the insertion of Tablets B until 160 min of the process, but gradually reduces thereafter, which is in good qualitative agreement with simulations.^3^ During previous coating trials that we conducted under the same process conditions, the minimum coating thickness of 30–40 μm that can be resolved with TPI was exceeded after approximately 80 min of process time. Because this corresponds to the total duration that Tablets B were coated for, we should not expect changes to the coating thickness on those tablets in our analysis. The coating thickness distribution during the first 80 min interval nevertheless appears to take the form of a Gaussian distribution centred around 40–50 μm. By introducing uncoated cores into an already coated batch of the same number of tablets, we speculate that it may take twice as long to exceed the minimum coating thickness that can be reliably resolved using TPI. With an increase in the total tablet population the number of tablet hits did not increase during the interval 140–160 min. Beyond this point, however, the number of tablet measurements remained relatively steady with increasing coating time. It should be noted that because the two coating thickness distributions are relatively broad, there is an overlap between the respective thickness distributions and therefore the tablet hits for Tablets A and B are approximated from the area underneath their respective distributions, whereas the figure for the total tablet hits is representative of the total number of measurements acquired. The initial sharp decline in the hit rate of Tablet A coincides with the insertion of the tablet cores and hence the corresponding increase in the hit rate for Tablet B (Figure 8). Following this initial change the hit rate for Tablet B remains relatively constant. The subsequent slow reduction in hit rate for Tablet A samples can be attributed to the particular selection criteria that were chosen for the detection of tablets with thinner coatings. Specifically, the thresholds used in the analysis were defined for thinly coated tablets only, so as the coating grows thicker the position and amplitude of the reflection peaks fall outside the detection thresholds for such tablets. An obvious way to alleviate this deficiency would be to define the thresholds on the basis of a worst case scenario, that is, thickest achievable coats. However by doing so, the accuracy of the measurements may become questionable as more coating reflections, not only just the normal reflections, may be permitted for thickness measurement. Clearly more work is needed in this regard to dynamically adjust the thresholds or define more than one set of selection criteria for each thickness population in order to capture high-quality reflections that would unveil more precise insights on the inter-tablet coating uniformities. However, the coating scenario tested in this run is completely artificial and extreme. During normal processing, such vast variation in coating thickness is highly unlikely.

### Further Deliberate Modifications of the Process Conditions

**Figure 9:**
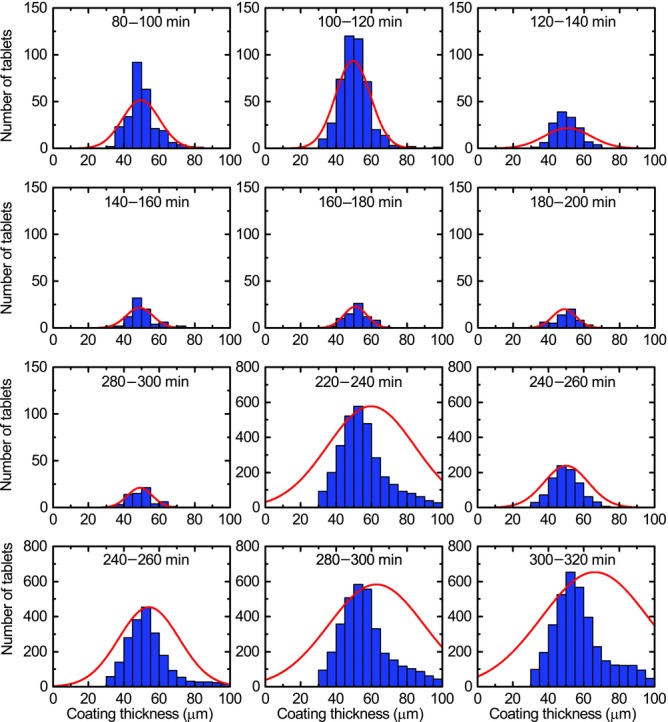
Histogram of tablet coating thickness measured inside the coating pan from 80 to 320 min process time. Intermittent disruptions to the spray rate were introduced in this coating run as described in the text.

**Figure 10:**
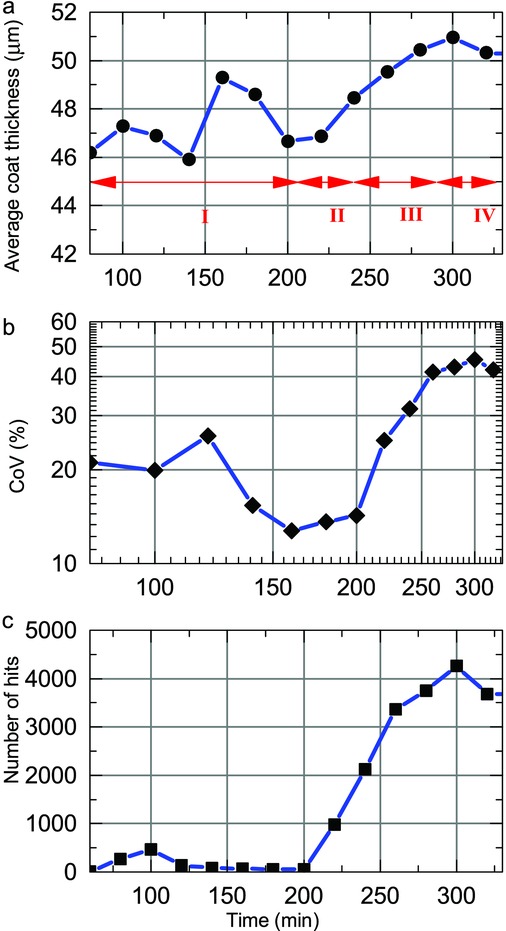
Curve-fitted mean coating thickness (a), inter-tablet variability (log–log scale) (b) and number of measurements (c) at different periods of the coating run that was characterised by process perturbations such as halting pan rotation (I), restart coating (II), reducing spray rate (III) and stop spraying (IV).

The ability of TPI to measure changes in coating thickness caused by changes in process conditions was further tested through additional coating trials conducted under non-standard, yet commonly encountered undesirable process conditions such as halting the coating pan, intermittent blockage of the spray guns and deliberate variation of spray rates. The measured thickness distributions after 80 min of coating are shown in Figure 9 (note the differences in relative hit rates in each 20 min interval). The best-fit Gaussian mean and variance of the thickness distribution are shown as a function of process time in Figure 10. During the 80–206 min period (Region I in Fig. 10) pan rotation was repeatedly halted for short periods, and spray was stopped for the intermittent cleaning of the spray guns (80–114 min). In the period from 114 to 206 min, the pan was set to jog with all spray operation stopped. The lack of pan rotation resulted in a localised and repeated measurement of a small sub-population of tablets leading to a relatively low and constant number of hits. As such, the thickness values derived during this period are not necessarily representative of the total tablet population. From 206 to 238 min (Region II) pan rotation and spray was restarted. A different coating colour was used subsequently (change from Acry-EZE pink to Acry-EZE white). As demonstrated previously, colour changes have little to no effect for the TPI coating thickness measurement as the optical properties at terahertz frequencies are not significantly affected by this change in pigment or lake as long as the overall bulk polymer of the coating formulation remains the same.^20^ In the following period, without any perturbation of the coating process, an increased number of measurements were acquired (reflected in the monotonic rise of the number of hits), and the measured coating thickness increased slightly (∼5 μm). The level of inter-tablet coating variability also increases monotonically in this period. During the 238–290 minutes period (Region III), the spray rate was reduced because of intermittent blockages in the spray guns, the effect of which can be observed in the slight decline of the rate of increase in mean coating thickness. This trend also appears to be replicated in the inter-tablet coating thickness variability. Finally, from 300 min onwards (Region IV), the mean thickness and variability plateaus. This coincides with the turning off of the spray guns in an effort to increase the exhaust temperature for the conclusion of the process at 330 min.

## CONCLUSIONS

In this study, we have outlined a systematic strategy to optimise the waveform selection algorithm for the coating thickness analysis using a TPI in-line sensor in order to account for the subtle non-concentricity of the coating pan. We have also demonstrated for the first time the use of an in-line terahertz sensor to study the effect of changes in the inter-tablet coating thickness distribution as the result of process variations during the tablet coating process. Our experimental results show that the removal of mixing baffles during the coating process will produce tablets with a higher level of coating thickness variation, evidently because of poorer mixing of tablets. Adding a batch of uncoated tablets during the coating operation resulted in the clear observation of two distinct thickness populations and demonstrated both the sensitivity and the robustness of the TPI technique for pharmaceutical coating process sensing. The effect of other process changes such as reducing the spray rate and halting the coating pan during the coating process was also demonstrated, and resulted in a clear measurement response of the terahertz in-line sensor. With the increased affordability of computational power, together with numerical modelling such as DEM, terahertz in-line sensor technology can play a vital role to unveil new insights into the film coating processes of pharmaceutical tablets. Such an understanding is critical to the successful development of high-quality advanced drug delivery systems such as active coatings and sustained-release coatings. At present it is the only technology that is capable of resolving inter-tablet coating variations *in situ* and in-line during production scale coating operations. Future work will aim to overcome the current limitation in minimum resolvable coating thickness of 30–40 μm by integrating optical coherence tomography with terahertz in-line sensing to further investigate the pharmaceutical film coating process.
